# Investigating the Feasibility of Epicranial Cortical Stimulation Using Concentric-Ring Electrodes: A Novel Minimally Invasive Neuromodulation Method

**DOI:** 10.3389/fnins.2019.00773

**Published:** 2019-07-24

**Authors:** Ahmad Khatoun, Boateng Asamoah, Myles Mc Laughlin

**Affiliations:** ^1^Research Group Experimental Oto-Rhino-Laryngology (ExpORL), Department of Neurosciences, KU Leuven, Leuven, Belgium; ^2^The Leuven Brain Institute, KU Leuven, Leuven, Belgium

**Keywords:** transcranial electrical stimulation, neuromodulation, concentric-ring electrode, motor cortex stimulation, direct cortical stimulation

## Abstract

**Background:**

Invasive cortical stimulation (ICS) is a neuromodulation method in which electrodes are implanted on the cortex to deliver chronic stimulation. ICS has been used to treat neurological disorders such as neuropathic pain, epilepsy, movement disorders and tinnitus. Noninvasive neuromodulation methods such as transcranial magnetic stimulation and transcranial electrical stimulation (TES) show great promise in treating some neurological disorders and require no surgery. However, only acute stimulation can be delivered. Epicranial current stimulation (ECS) is a novel concept for delivering chronic neuromodulation through subcutaneous electrodes implanted on the skull. The use of concentric-ring ECS electrodes may allow spatially focused stimulation and offer a less invasive alternative to ICS.

**Objectives:**

Demonstrate ECS proof-of-concept using concentric-ring electrodes in rats and then use a computational model to explore the feasibility and limitations of ECS in humans.

**Methods:**

ECS concentric-ring electrodes were implanted in 6 rats and pulsatile stimulation delivered to the motor cortex. An MRI based electro-anatomical human head model was used to explore different ECS concentric-ring electrode designs and these were compared with ICS and TES.

**Results:**

Concentric-ring ECS electrodes can selectively stimulate the rat motor cortex. The computational model showed that the concentric-ring ECS electrode design can be optimized to achieve focused cortical stimulation. In general, focality was less than ICS but greater than noninvasive transcranial current stimulation.

**Conclusion:**

ECS could be a promising minimally invasive alternative to ICS. Further work in large animal models and patients is needed to demonstrate feasibility and long-term stability.

## Introduction

Electrical and magnetic brain stimulation can successfully treat a wide range of neurological and psychiatric disorders. In general, these neuromodulation methods fall into two categories: invasive or noninvasive. Invasive neuromodulation methods, such as deep brain stimulation (DBS) and invasive cortical stimulation (ICS, often simply referred to as motor cortex stimulation), require the implantation of an electrode array in a specific brain area to deliver chronic electrical stimulation. The advantage of invasive neuromodulation is that relatively strong stimulation can be chronically delivered to a very focused target. The main disadvantage is the highly invasive nature of the implantation procedure: a burr hole or craniotomy is required and the patient is often awake during parts of the surgery to ensure correct electrode placement. This surgical procedure exposes the patient to significant risk and discomfort and increases therapy cost. Noninvasive neuromodulation methods such as transcranial magnetic stimulation (TMS), transcranial direct current stimulation and transcranial alternating current stimulation (both referred to as transcranial electrical stimulation or TES) have the advantage that no surgery is required, thus significantly reducing patient risk and discomfort, in addition to reducing costs associated with hospitalization and surgery. However, noninvasive neuromodulation methods have a number of significant disadvantages: Stimulation can only be delivered in an acute clinical or laboratory setting. The neuromodulatory effects of TES are typically relatively weak and not well focused. While the effects of TMS are stronger and more focused, it requires expensive and bulky equipment to generate the strong magnetic fields required.

Recently, a novel minimally invasive approach to neuromodulation has emerged – epicranial current stimulation (ECS). In ECS an electrode array is implanted under the scalp and on, or in close proximity to, the skull. ECS has a number of potential advantages over standard invasive and noninvasive neuromodulation methods. The implantation of an ECS device is much less invasive than an ICS or DBS device and could potentially be done under local anesthesia. This would allow delivery of chronic stimulation at a significantly reduced cost, in addition to reduced patient risk and discomfort. With TES most of the delivered current is shunted by the scalp, resulting in relatively weak neuromodulatory effects. By stimulating under the skin, ECS could potentially deliver much stronger neuromodulation. ECS has been tested in animal models and shown to be an effective method for controlling epileptic seizures (Besio et al., 2007; Berényi et al., 2012; Besio et al., 2013). Beyond epilepsy, ECS could offer an alternative approach to treating the wide range of neurological and psychiatric disorders that are currently treated with standard neuromodulation methods. For example ICS is used to treat neuropathic pain (Tsubokawa et al., 1991; Tsubokawa et al., 1993; Nguyen et al., 2000) and has been investigated as a treatment for movement disorders (Pagni et al., 2005; Canavero and Bonicalzi, 2007; Priori and Lefaucheur, 2007; Moro et al., 2011) and depression (Nahas et al., 2010; Kopell et al., 2011). ECS has the potential to offer a less invasive neuromodulation therapy for these disorders. ECS has not yet been tested in humans but systems for patient use are currently in development (Lee et al., 2007).

The aim of the current study was to investigate the feasibility and limitations of using concentric-ring electrodes for ECS in humans. Concentric-ring electrodes consist of an inner disk electrode surrounded by an outer ring and have the potential to deliver more focused stimulation than standard mono or bipolar electrode configurations (Datta et al., 2008; Bortoletto et al., 2016; Gbadeyan et al., 2016; Heise et al., 2016; Martin et al., 2017). We first tested the feasibility of using ECS concentric-ring electrodes in a rat experiment. We verified that we could achieve selective stimulation of the motor cortex by measuring stimulation induced limb movements and comparing the results to that of unfocused stimulation. We then used an MRI based electro-anatomical human head computational model to simulate the electric field strength and focality that could be achieved in patients with ECS concentric-ring electrodes. We used the model to investigate the effect of different ECS concentric-ring electrode designs on electric field strength and focality. Finally, we compared the strength and focality of the cortical electric field generated by ECS with that generated by both ICS and TES.

## Materials and Methods

### Concentric-Ring ECS: Proof-of-Concept in Rats

#### Animals

Six male Wistar rats (391 ± 91 g, Janvier labs, France) were used. They were housed in a rat colony at ∼19^∘^C and maintained on a 14/10 h light/dark cycle (lights on at 7:00 a.m.). Rats had unrestricted access to food and water. All procedures were approved by the KU Leuven ethics committee for laboratory experimentation (project P096/2015).

#### Surgery and Preparation

On experiment days rats were anaesthetized with an IP injection of a combination of ketamine (45 mg/kg, Anestekin, Eurovet, Belgium) and medetomidine HCl (0.3 mg/kg, Narcostart, Kela Veterinaria, Belgium), placed in a stereotaxic frame (Narishige type SR-6, No. 7905) on a heating pad and the core temperature monitored via a metal rectal probe. Anesthesia level was routinely monitored using the toe-pinch reflex. The anesthesia level was held constant by giving an additional IP injection of around 100 μL of the ketamine-medetomidine mixture approximately every hour. The skull was exposed by making an incision and then retracting the scalp. A tripolar concentric-ring electrode (CRE medical, Kingston, United States, outer ring diameter: 5.5 mm, inner ring diameter 5 mm and center disk diameter 2 mm) was used to target the hind-limb area of the motor cortex. The general location was determined stereotactically using coordinates from the Paxinos and Watson rat brain atlas (Paxinos and Watson, 2007). The specific location was then found by slowly moving the electrode while delivering electrical stimulation. Using this approach the electrode was finally positioned on the skull over the motor cortex at a location that could elicit a limb movement.

#### Electrical Stimulation

Electrical stimulation was delivered using a DS5 current source (Digitimer, Hertfordshire, United Kingdom) controlled by an analog voltage waveform input. The voltage waveform was generated using an output channel on a data acquisition card (NI USB-6343, National Instruments, TX, United States) and controlled via custom written MATLAB software (MathWorks, MA, United States) at a sample rate of 20 kHz. Electrical stimulation was delivered through the central disc of the concentric-ring electrode and returned through the outer ring. Stimulation consisted of biphasic rectangular pulses (300 μs per phase) delivered in a pulse train (10 pulses per train, 300 pulses per second) and repeated every one second. These parameters were already shown to induce measurable and reproducible kick-like limb movements (Khatoun et al., 2017). We measured the limb movement when the stimulation amplitude was increased from 1 to 8 mA in 1 mA steps while keeping all other parameters the same. This stimulation amplitude range is enough to cover the variability in the limb movement threshold that occurs between the different rats. The complete sweep (i.e., all amplitudes from 1 to 8 mA) was repeated four times with a 1 min break between repetitions. To deliver unfocused (or monopolar) stimulation, the concentric-ring electrode was kept in the same location. However, now stimulation was only delivered through the central ring. No current was returned through the outer ring, instead current was returned through a large disk electrode (9 mm diameter) placed on the midline 9 mm posterior to bregma. For unfocused electrode configuration, stimulation parameters were exactly the same, except that the stimulation amplitude was increased from 1 to 4 mA. For the same current amplitude, an unfocused electrode configuration gives a stronger electric field, meaning that lower current amplitudes are needed to cause a limb movement.

#### Limb Movement Measurements and Quantification

To monitor the limb movement two tri-axial accelerometers (ADXL353, Analog Devices, MA, United States) were used. One accelerometer was attached to the targeted limb contralateral to the stimulation site and the other was attached to another limb of interest. Comparing data from both accelerometers showed that we selectively stimulated the motor area controlling the targeted limb, while avoiding stimulation of the motor area controlling the other limb. The six axes (three from each accelerometer) were digitized (NI USB-6216, National Instruments) at 4 kHz sample rate, displayed and recorded for off-line analysis using custom written MATLAB software (MathWorks, MA, United States).

After the experiment, the raw acceleration data were band pass filtered between 3 and 500 Hz (second-order Butterworth) and integrated twice to give the limb displacement in arbitrary units. Principal component analysis was used to combine the three displacement axes and limb displacement was defined as the first principal component. The difference between the minimum and maximum limb displacement occurring after stimulation was then calculated to give limb displacement amplitude for each stimulus presentation.

### Concentric-Ring ECS: Feasibility in Humans

An electro-anatomical human head computational model was used to investigate the feasibility of applying ECS in humans and to explore possible electrode designs. The anatomical model enabled us to obtain a quantitative estimate of electric field strength in the different tissue layers during ECS and to explore the effect of different electrode designs. Additionally, we used the model to compare the results with other electrical neuromodulation techniques such as ICS and TES.

#### MIDA Anatomical Model

The model is based on modified data from the MIDA study: a publicly available homogenous head model, which was built by combining different tissue classes from a multimodal imaging–based detailed anatomical (MIDA) model of human head and neck (FDA, Center for Devices and Radiological Health, MD, United States, and IT’IS Foundation, Zurich, Switzerland) (Iacono et al., 2015). The MIDA model was imported into ScanIP 7 (Simpleware Ltd., Exeter, United Kingdom) as a series of 116 surface meshes – each mesh representing a different tissue type. We first simplified the model by reducing it to tissue types relevant for this study and with known conductivity values. To do this we converted the meshes to volumes (masks). Then merged tissue volumes to obtain just five tissue types and assigned them the following standard electrical conductivity values (σ): skin 0.465 S/m; skull 0.01 S/m; CSF 1.65 S/m; gray matter 0.27 S/m; and white matter 0.126 S/m (Peters et al., 2001; Akhtari et al., 2002; Datta et al., 2009; Gabriel et al., 2009).

#### Addition of ECS With Concentric-Ring Electrode to Anatomical Model

ECS concentric-ring was modeled as an inner disc electrode and an outer ring electrode both embedded in an insulating silicon material (polydimethylsiloxane or PDMS, typically used for invasive electrode designs) (Meacham et al., 2008; Guo et al., 2013; Ochoa et al., 2013; Salam et al., 2014). The electrode was placed in contact with the skull with the disc and ring electrodes facing the skull and the silicon layer in contact with the skin. We assumed that the electrode pushed the overlaying skin tissue resulting in a slight skin bulge. This was modeled by dilating the skin layer above the electrode with similar dimensions to the electrode. The edges of the dilation were further smoothed to mimic skin stretch. The silicon layer was modeled as a subcutaneous 1.6 mm thick layer of polydimethylsiloxane (PDMS) with 10^–14^ S/m conductivity (Mark, 1999; Danial et al., 2017), with a radius of 80 mm.

#### Effect of ECS Concentric-Ring Electrode Design

We used the model to explore the effect of two ECS concentric-ring electrode design parameters: (1) the spacing between the central disc electrode and the outer ring electrode (referred to as disc-ring spacing) and (2) the size of the central disc electrode (referred to as disc size). Both parameters are expected to effect the strength and focality of the electric field reaching the cortex. The central disc was always positioned in the center of the same silicon layer. Firstly, we fixed the central disc to have a diameter of 8 mm (50.25 mm^2^ surface area) and investigated the effect of disc-ring spacing using three settings for the ring electrode diameters: close, standard and far. Table 1 provides the ring diameters. Secondly, we investigated the effect of the disc size using three sizes for the central disc: small, medium and large. Table 1 provides the disc diameters and surface areas.

**TABLE 1:** List of the ECS concentric-ring electrode dimensions investigated in the study.

	**Disc**-**ring spacing**			**Central disc size**		
	**Far**	**Standard**	**Close**	**Large**	**Medium**	**Small**
Central electrode diameter (mm)	8	8	8	16	8	4
Ring electrode diameters (mm)	Inner: 52 Outer: 55.8	Inner: 26 Outer: 33	Inner: 13 Outer: 24.1	Not applicable	Not applicable	Not applicable

For a concentric-ring electrode, the central disc is the stimulating electrode and the current returns via the outer ring electrode. This was the standard configuration used in the model and was the configuration used to test the effect of disc-ring spacing. However, since changing the size of the central disc will also change the disc-ring spacing, we opted to use a monopolar stimulation configuration (i.e., where current returns via an implantable pulse generator located in the chest) to investigate the effect of disc size (i.e., small, medium, and large) in isolation. The MIDAS model contains the head and the neck only and does not contain a body. We modeled the body as a cube connected to the neck (135 mm × 30 mm × 100 mm dimension) and we set the bottom surface of this cube as the return electrode (135 mm × 100 mm). The chosen dimension of the body cube provides a compromise solution between computational cost and reality. Importantly, we choose a body size that completely covered the base of the neck which closely matches the real life situation. This ensures that current flow patterns are only minimally affected as they passed from the neck to the body, thus current flow patterns in the brain will also be relatively unaffected. With this approach increasing the body size would only have a minimal effect the electric field in the brain.

#### Comparison With ICS and TES

To compare ECS with other invasive and noninvasive neuromodulation methods, the same model was used to simulate both ICS and TES. Given its invasive nature and its direct intact with the cortex, we would expect ICS to induce a stronger and more focused cortical electric field than ECS. On the other hand, we would expect TES to induce weaker and less focused cortical field than ECS given that most of the current is shunted by skin during TES.

The ICS electrode was modeled as a 3.3 mm diameter disk electrode (Lesser et al., 2010) with thickness of 1.6 mm (Kim et al., 2011). ICS used the same monopolar configuration described above.

The TES electrode was modeled as a central disc and ring electrode configuration. The central electrode diameter was set to be 16 mm and the ring electrode’s inner and outer diameters were set to be 52 and 66 mm, respectively. These dimensions are similar to those reported in TES concentric-ring electrode studies (Datta et al., 2008; Gbadeyan et al., 2016; Heise et al., 2016; Martin et al., 2017). Each TES electrode (disc and ring) were modeled as a 1.6 mm thick layer of gel with 0.3 S/m conductivity in direct contact with the scalp.

One gyral crown was manually selected from the motor cortex and the central electrodes from all the methods (ECS, ICS, and TES) were positioned rectilinearly above the same gyral crown.

#### Electric Field Calculation

In ScanIP, volumetric tetrahedral models were calculated for all generated models. The results were imported into COMSOL multiphysics 5.3 (COMSOL, Inc., Burlington, MA, United States) where electric field (E) and current density (J) was calculated by solving Laplace’s equation,

(1) $$\begin{array}{c} \nabla \cdot \sigma \nabla \varphi =0\\ E=|\nabla \varphi |\\ J=\sigma |E| \end{array}$$

with φ representing the electrical potential. This assumes a quasi-static approximation of Maxwell’s equations, valid for alternating electric fields in the brain with frequencies < 1 MHz (Nunez and Srinivasan, 2006). Boundary conditions were set to have a positive current at the anodic central electrode with peak-amplitude equal to 1 mA and the negative current was set on the ring electrode during ECS and TES and on the bottom area of the modeled body during monopolar ICS.

To avoid a measure of maximum electric field strength that is skewed by one or two voxels containing very high values, the maximum electric field strength was calculated as the average value of the electric field strength in a 10 mm^3^ volume containing the highest electric field strengths in one particular tissue. This 10 mm^3^ volume was found by first ranking all voxels in one tissue from high to low electric field and then selecting the number of voxels, starting with the highest ranking and progressing to lower, which were needed to make up a 10 mm^3^ volume. To quantify electric field spatial spread (i.e., a measure of focality) we calculated the half-value volume (Deng et al., 2013; Khatoun et al., 2018), this is the volume of the brain with an electric field magnitude higher than half of the maximum electric field strength. For a field that is distributed over a larger volume of brain, the half-value volume will be higher than for a field that is distributed over a smaller volume of brain.

## Results

### Concentric-Ring ECS: Proof-of-Concept in Rats

**FIGURE 1:**
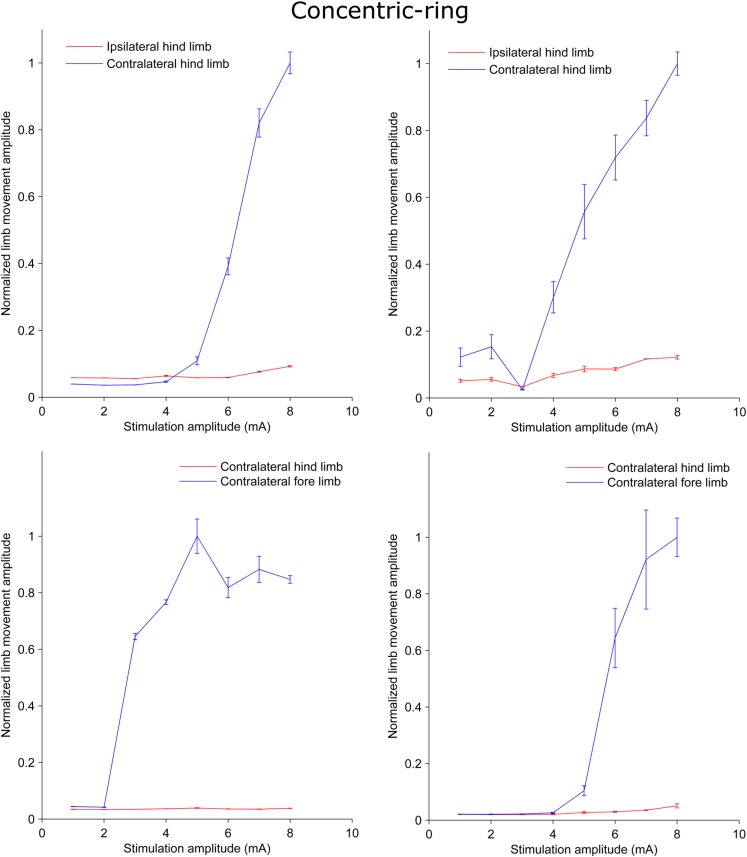
Plots from rat experiments showing the limb movement amplitude as a function of stimulation amplitude applied to concentric-ring ECS electrode placed over the rat motor cortex. The upper panel compares the movement in the hind limbs while the lower panel compares the movement in the hind limb to that in the fore limb. Error bars represent the standard deviation. When stimulation targeted one of the hind limbs and the amplitude was below threshold (upper panel), both the hind limbs ipsilateral (red) and contralateral (blue) to the side of stimulation showed no increase in the movement amplitude. However, when stimulation amplitude was increased above threshold there was significant increase in the contralateral limb movement and no, or relatively low, ipsilateral movement. Similar results were obtained in the lower panel. However, this time stimulation targeted the fore limb cortical area. Movement was detected in the contralateral fore limb (blue) but not in the contralateral hind limb (red). This indicates that concentric-ring ECS can cause selective stimulation of the rat motor cortex.

**FIGURE 2:**
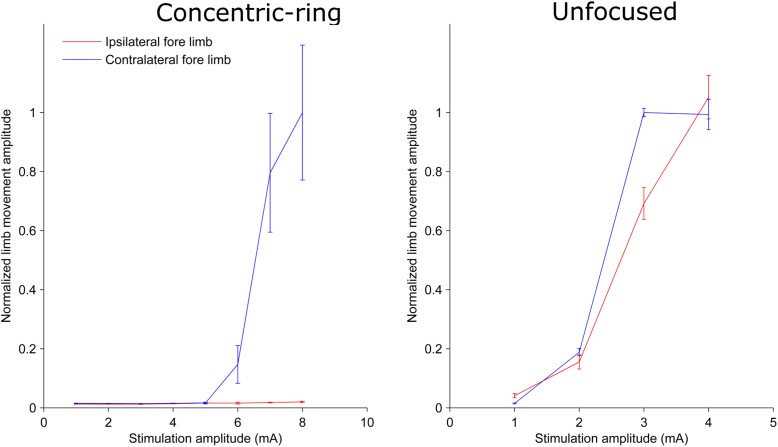
Plots from one rat showing the limb movement amplitude as a function of stimulation amplitude using a concentric-ring ECS **(left panel)** and unfocused ECS **(right panel)**. Error bars represent the standard deviation. Note the difference in the stimulation amplitude scale between the two graphs. The concentric-ring electrode shows a selective stimulation of the contralateral fore limb (blue) with a threshold of 6 mA. On the other hand, the unfocused stimulation shows a movement in both contralateral (blue) and ipsilateral (red) fore limbs with a threshold of 2 mA. This indicates that the concentric-ring electrode provides stimulation of one specific target brain area which is not possible with an unfocused ECS electrode. However, because focused electrode produce a weaker electric field in the brain (see Figure 4), the concentric-ring ECS has a higher threshold than unfocused ECS.

We used a rat experiment to demonstrate that ECS with concentric-ring electrodes can deliver focused cortical stimulation, strong enough to cause selective limb movement. The left upper panel in Figure 1 is an example from one rat showing the amplitude of the hind limb movement as a function of the pulse-train amplitude delivered through an ECS concentric-ring electrode. When stimulation amplitude was below 4 mA, neither of the hind limbs contralateral (blue) nor the ipsilateral (red) to the stimulated hemisphere moved. When the stimulation amplitude was increased above 4 mA and up to 8 mA, the contralateral hind limb showed a corresponding increase in movement amplitude. However, the ipsilateral hind limb did not move, even at these higher amplitudes. The right upper panel in Figure 1 shows the results from a second rat. We observed a similar effect to that of the first rat. However, the threshold for limb movement in this rat was slightly lower at around 3 mA. At higher amplitudes we also observed a small increase in the ipsilateral hind limb movement, but this was much smaller than in the contralateral hind limb. The lower panel of Figure 1 shows similar results to the upper one but from different rats. However, this time stimulation targeted the fore limb cortical area. Movement was detected in the contralateral fore limb but not in the contralateral hind limb. The threshold for limb movement in these rats were 3 and 5 mA, respectively. Similar results to the panels in Figure 1 were obtained from seven limbs recorded from three different rats (Supplementary Table 1 and Supplementary Figures 1–4). In two of these rats we compared hind and fore limbs movements from the same side (see Supplementary Figures 1, 2). The results from these measurements show a movement of the targeted contralateral limb but not the other contralateral limb. Interestingly, we compared the focality of concentric-ring to unfocused stimulations in these three rats. Figure 2 shows an example from one rat. The left panel shows the amplitude of the hind limbs movements when the concentric-ring electrode was used and the right panel shows the movements amplitudes of the same limbs when the unfocused electrode was used. The results show that using concentric-ring electrode the stimulation was focused to the contralateral hind limb with threshold of 6 mA while no movement was recorded in the ipsilateral hind limb even at 8 mA. On the other hand, during the unfocused stimulation, the ipsilateral hind limb showed a high but slightly lower limb movement compared to the contralateral limb. In addition, the threshold for limb inducing limb movement was 2 mA which is lower than that of the concentric-ring electrode. Thus, results from the rat experiment show that ECS with concentric-ring electrodes can cause relatively strong, yet selective (i.e., focused), neuromodulation of the rat motor cortex. However, given the large differences in head size, skull thickness and morphology between rats and humans, it was unclear if ECS with concentric-ring electrode in humans would also be feasible.

### Concentric-Ring ECS: Feasibility in Humans

#### Exploring the Feasibility of ECS

**FIGURE 3:**
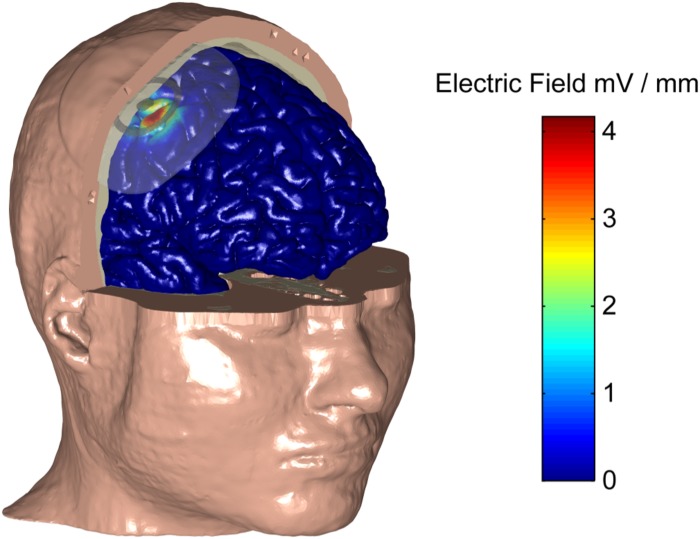
A human head electro-anatomical computational model was used to illustrate the concept of ECS with a concentric-ring electrode. Part of the skin and skull have been made transparent to reval the ECS concentic-ring electrode, which consists of a central disc and outer ring electrode (gray) embedded in an insulating silicone layer (partially transparent). The disc and ring electrode are in contact with the skull but insulated from the skin by the silicone layer. The CSF is not shown for simplicity. The electric field strenght on the cortical surface (see color bar) caused by a 1 mA current applied through the ECS electrode is shown. Using this electrode design, 1 mA ECS creates a relatively focused electric field in the cortex with the maximum electric field strength of 3.82 V/m.

We used an electro-anatomical human head computational model to investigate the feasibility of applying ECS in humans and to explore different electrode designs. Figure 3 shows a rendered representation from the computational model to illustrate the concept of ECS with a concentric-ring electrode. The electrode consists of a central disc and outer ring electrode (gray) embedded in a silicone layer (partially transparent). The disc and ring electrode are in contact with the skull but insulated from the skin by the silicone layer. Here, the medium disc size was used with the standard spacing (see Table 1). We applied a 1 mA current through the ECS electrode and calculated the electric field strength in each tissue layer. In Figure 3 the electric field strength is color encoded on the cortical surface. It shows that ECS with a concentric-ring electrode generates a relatively focused electric field in the cortex. For 1 mA ECS using these electrode dimensions the maximum electric field strength in the cortex was 3.82 V/m.

#### Effect of ECS Concentric-Ring Electrode Design

**FIGURE 4:**
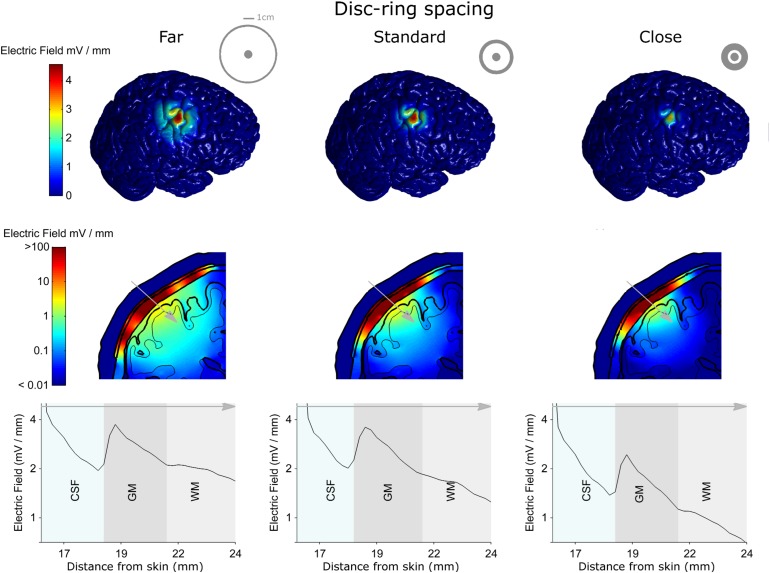
The electro-anatomical head model was used to investigate the effect of disc-ring spacing on the focality and strength of the electric field reaching the cortex. The left, central and right columns show model data for the far, standard and close disc-ring spacing respectively (shown as insets in the top row), when the same 1 mA current is delivered through each spacing. The top row shows how reducing the disc-ring spacing causes a more focused electric field on the brain. However, as highlighted in the second and third rows this comes at the cost of reducing the electric field strength. Thus, when keeping current constant, there is always a trade-off between electric field strength and focality. A 2-dimensional coronal, partial, cross-section is shown in the middle row using a logarithmic color scale. The different model tissues are outlined in black – skin, skull, CSF, gray matter (GM) and white matter (WM). A 1-dimensional plot of the electric field strength along the position indicated by the gray arrow is shown on the bottom row. This shows how the electric field magnitude decreases with distance from the electrode, how it is affected by each tissue type (only CSF, GM, and WM shown for a clearer comparison) and by the disc-ring spacing. Note that the maximum electric field values reported in the manuscript are based on the 3-dimentional brain volume (first row) and not on the 2-dimentional (second row) or the 1-dimensional (third row) as they represent values being interpolated from the 3-dimentional data.

Next, we used the model to test the effect of disc-ring spacing on the electric field distribution. Figure 4 shows the results for three different spacing between the central and the ring electrode: Far, standard and close (left, middle, and right column, respectively, inner ring dimensions: 52, 26, and 13 mm, respectively). The upper row in Figure 4 shows the electric field strength generated at the cortical surface when a 1 mA current was applied through each of the electrodes. The second row shows a 2-dimensional cross-section from the same models; while the third row shows the electric field strength along a 1-dimensional line indicated by a gray arrow on the cross-section. The far spacing showed a broad electric field distribution with a half-value volume of 169.44 mm^3^ and a maximum electric field strength of less than 4.13 V/m. Moving to the standard spacing (i.e., ring closer to the disc) resulted in a more focused but weaker field with values of 103.60 mm^3^ and 3.82 V/m for the half-value volume and the maximum electric field strength, respectively. Reducing the spacing further to the close setting resulted in a more focused but even weaker field with values of 55.63 mm^3^ and 2.70 V/m for the half-volume percentage and the maximum electric field strength, respectively. These values, along with the electric field strength in the skin and skull are summarized in Table 2. Thus, when keeping the current constant, changing disc-ring spacing causes a trade-off between electric field strength and focality.

**TABLE 2:** List of the maximum electric field strengths (E, V/m) obtained in each tissue type (skin, skull, and brain) for each of the three disc-ring spacing settings (close, standard, and far) and disc size settings (small, medium, and large) investigated.

**Stim Amp**			**1 mA**			**2 mA**			**10 mA**		
**Max E in tissue (V/m)**			**E_skin_**	**E_skull_**	**E_Brain_**	**E_skin_**	**E_skull_**	**E_Brain_**	**E_skin_**	**E_skull_**	**E_Brain_**
**Disc-ring spacing**		Far	0.210	2.225 × 10^3^	4.13	0.420	4.450 × 10^3^	8.26	2.10	2.225 × 10^4^	41.3
		Standard	0.020	2.229 × 10^3^	3.82	0.040	4.458 × 10^3^	7.64	0.200	2.229 × 10^4^	38.2
		Close	0.005	2.290 × 10^3^	2.70	0.010	4.450 × 10^3^	5.40	0.050	2.290 × 10^4^	27.0
**Disc size**		Large	0.175	839	2.86	0.350	1678	5.72	1.750	8390	28.6
		Medium	0.175	2.224 × 10^3^	4.16	0.350	4.448 × 10^3^	8.32	1.750	2.224 × 10^4^	41.6
		Small	0.175	4.942 × 10^3^	4.84	0.350	9.884 × 10^3^	9.68	1.750	4.942 × 10^4^	48.4
	ICS		0.21	15.80	42.52						
	TES		32.80	11.64	0.11						

*Equivalent values for ICS and TES are shown. The effect of increasing the current delivered through the ECS concentric-ring electrodes to 2 and 10 mA are also shown.*

**FIGURE 5:**
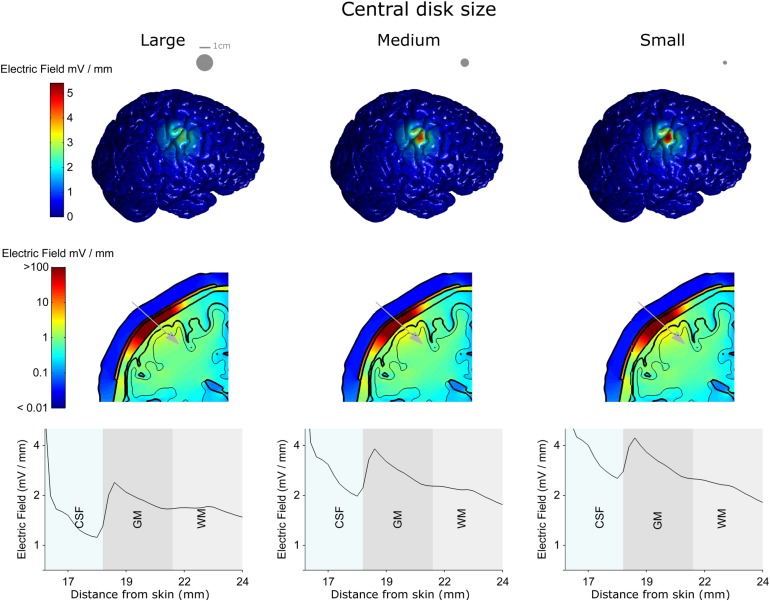
The electro-anatomical head model was used to investigate the effect of disc size on the focality and strength of the electric field reaching the cortex. The left, central and right columns show model data for the large, medium and small disc sizes respectively, when the same 1 mA current is delivered through each spacing. Note – a monopolar configuration was used with these simulations. The top row shows how reducing the disc size (shown as insets in the top row) causes a more focused electric field on the brain. As highlighted in the second and third rows the smaller disc size also causes a stronger electric field in the brain. However, the smaller disc size increases the current density under the electrode (see text). Thus, there is always a trade-off between electric field strength and focality. A 2-dimensional coronal, partial, cross-section is shown in the middle row using a logarithmic color scale. The different model tissues are outlined in black – skin, skull, CSF, gray matter (GM) and white matter (WM). A 1-dimensional plot of the electric field strength along the position indicated by the gray arrow is shown on the bottom row. This shows how the electric field magnitude decreases with distance from the electrode, how it is affected by each tissue type (only CSF, GM, and WM shown for a clearer comparison) and by the disc size.

Figure 5 shows the results for three different central disc sizes: large, medium and small (left, middle, and right column, respectively, disc diameters: 16, 8, and 4 mm) all with a 1 mA current amplitude. Note, that a monopolar configuration was used for all three. The large disc electrode showed a broad electric field distribution with a half-value volume of 646.81 mm^3^ and a maximum electric field strength of less than 2.86 V/m. Reducing the central electrode size to medium resulted in a more focused and stronger field with values of 190.18 mm^3^ and 4.16 V/m for the half-value volume and the maximum electric field strength, respectively. Reducing the central electrode size to small resulted in a more focused field with values of 118.70 mm^3^ and 4.84 V/m for the half-volume percentage and maximum electric field strength, respectively. These values, along with the electric field strength in the skin and skull are summarized in Table 2. Thus, reducing disc size produces a stronger and more focused electric field in the brain. However, the trade-off here is with current density at the electrode-skull interface and electrode impedance. Reducing the disc size increases the current density at the electrode surface and increases the electrode impedance. For a current of 1 mA the small, medium and large disc sizes had current densities of 0.080, 0.020, and 0.005 mA/mm^2^, respectively.

#### Comparison With ICS and TES

**FIGURE 6:**
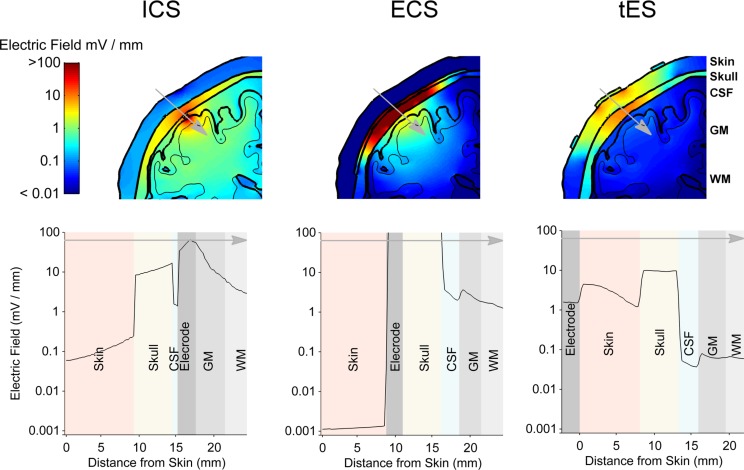
The results from the electro-anatomical computational model comparing the electric field distribution in different tissues resulting from a 1 mA ECS to that of ICS and TES. A 2-dimensional coronal, partial, cross-section is shown in the upper row with the color scale being logarithmic. The different tissues represented in the model are indicated – skin, skull, CSF, gray matter (GM) and white matter (WM). A 1-dimensional plot of the electric field strength along the position indicated by the gray arrow is shown in the lower row. This shows how the electric field magnitude decreases with distance from the electrode and how it is affected by each tissue type. Again, note the logarithmic scale on the axis showing the electric field. ECS shows a more focused and stronger cortical field than TES, but less focused and weaker cortical field than ICS.

To put the potential neuromodulatory effects of ECS with concentric-ring electrodes into perspective we used the same model to simulate more standard neuromodulation methods, namely ICS and TES. Figure 6 shows the model results comparing the electric field strengths generated in each tissue for the three neuromodulation methods, when the same 1 mA current was applied (from left to right: ICS, ECS with standard disc-ring spacing and medium disc size, TES). The upper row shows a 2-dimensional coronal cross-section passing through the electrode center for each neuromodulation method. The lower row shows the corresponding 1-dimensional plot of the electric field strength along the position indicated by the gray arrow in the upper panels. The figure highlights how the electric field magnitude decreases with distance from each electrode type and how the electric field is affected by the different tissues. Note, the same logarithmic scale is used to compare the electric fields across all plots. The results show that for a 1 mA current, ICS induced the strongest cortical field with the maximum electric field strength of around 42.52 V/m. For the same current amplitude, ECS showed maximum electric field strength in the cortex of 3.82 V/m with high electric fields values in the skull (greater than 100 V/m) and low field strengths in the skin with the maximum electric field strength of 0.02 V/m. As expected, TES showed the weakest cortical field and the highest fields in the skin with approximate maximum electric field strength of 0.11 and 32.8 V/m, respectively. In terms of cortical fields spatial distribution, ICS caused the most focused stimulation with the half-value volume of 5.64 mm^3^ followed by ECS with a value of 103.60 mm^3^ and then TES with a value of 2138.10 mm^3^. Our estimated cortical electric fields are in agreement with other tES modeling studies (Datta et al., 2008; Bortoletto et al., 2016; Nikolin et al., 2019). The validity of such models has already been confirmed using invasive recordings (Lafon et al., 2017; Vöröslakos et al., 2018). In summary, the model predicts that both electric field strength and focality will be reduced by one order of magnitude when moving from the invasive ICS to the minimally invasive ECS. Then, the focality will be reduced again by another order of magnitude when going from ECS to the noninvasive TES. Thus, when the delivered current is held constant, there is a clear trade-off between the degree of invasiveness and the strength and focality of the electric field than can reach the brain.

#### Increasing ECS Stimulation Amplitude

With ECS, it may be possible to increase the strength of the electric field reaching the brain by increasing the current amplitude. To investigate the feasibility and limitations of achieving stronger electric fields in the cortex with concentric-ring ECS we simulated the effect of increasing the current amplitude delivered through the ECS electrode. This is a simple exercise, given that the model is completely linear. However, when taken in context with the ICS and TES models, the results give important insight into the potential strength of the neuromodulatory effects that could be achieved with ECS. Table 2 shows the maximal electric field strengths in the skin, skull and cortex for a range of concentric-ring electrode designs when a 1, 2, or 10 mA current is delivered. These are compared with 1 mA TES and 1 mA ICS. Increasing the ECS current amplitude to 10 mA allows delivery of electric field strengths to the brain which are in the same range as ICS. However, even with the concentric-ring design, these fields are still less focused that those achievable with ICS.

## Discussion

We first evaluated ECS using concentric-ring electrodes in a rat motor cortex stimulation experiment. We demonstrated that concentric-ring ECS can produce strong and focused neuromodulation: stimulation was strong enough to cause a limb movement and focused enough to cause movement in only the target limb. This is in contrast to unfocused stimulation which always showed a movement in more than one limb. However, it is difficult to directly translate current amplitude thresholds and electric field strengths from the rat brain to the human. Therefore, we then used an electro-anatomical human head model to explore the feasibility of using concentric-ring ECS in patients. We showed that depending on the electrode design, a 1 mA current delivered through an ECS concentric-ring electrode would cause a relatively focused electric field of between 2.70 and 4.13 V/m in the cortex. To put the potential neuromodulatory effects of concentric-ring ECS into context we used the same model to simulate ICS and TES. These are both standard neuromodulation methods where the effects of the electric field strengths and effects are reasonable well known. We showed that for the equivalent current amplitude, concentric-ring ECS could produce cortical electric fields that are an order of magnitude stronger and more focused than TES. However, for an equivalent current amplitude, ECS fields are an order of magnitude lower and less spatially focused than those achieved with ICS. Increasing ECS current amplitude to 10 mA brings the electric field strength into the same range as 1 mA ICS, but this is at the cost of stronger electric fields in the skull and skin. Within the context of these results, we now discuss potential applications for concentric-ring ECS.

### Potential Applications

ECS has the potential to deliver much stronger neuromodulation than is achievable with TES, in addition to potentially delivering continuous stimulation. This could be of great value, particularly given the recent controversies in the TES field concerning: (1) the weak electric field strength in the cortex (Huang et al., 2017; Lafon et al., 2017); (2) the ongoing debate around the potential absence of neuromodulatory effects in some protocols (Lafon et al., 2017); and (3) the potential role of transcutaneous stimulation of peripheral nerves in the scalp in mediating TES effects (Asamoah et al., 2019). Our results using concentric ring electrode show that 1 mA of ECS produces an electric field in the skin that is more than three orders of magnitude weaker than that induced during 1 mA of TES. In addition, increasing the stimulation amplitude during ECS to 10 mA still induces an electric field in the skin that is much weaker than the threshold to fire an action potential in the peripheral nerves (0.2 V/m compared to 4–6 V/m) (So et al., 2004). These results indicate that it would require more than 100 mA of ECS current, using the concentric-ring electrode, before subjects perceive the stimulation in the skin. However, the potential increase in neuromodulation strength of ECS over TES comes at the cost of moving from a noninvasive to a minimally invasive method. Therefore, ECS applications are likely to be the treatment of neurological or psychiatric disorders that are severe enough to merit surgical intervention such as medically refractory epilepsy or neuropathic pain, advanced stage movement disorders, or treatment-resistant major depression. A wide range of studies have already shown that invasive neuromodulation methods such as ICS (Tsubokawa et al., 1991; Tsubokawa et al., 1993; Nguyen et al., 2000) and DBS (Benabid et al., 1991; Blond and Siegfried, 1991; Bewernick et al., 2010; Zhang et al., 2010; Volkmann et al., 2012) can be used to treat each of these conditions. Thus, for brain disorders that are already treated using invasive neurosurgical approaches, ECS may offer a less invasive alternative. The main advantage over ICS or DBS would be a much shorter and less invasive surgical approach, which could be performed under local anesthesia, thus reducing cost, risk and patient discomfort. One disadvantage of ECS over ICS, is that for the same 1 mA current, ECS will provide a much weaker and less focused neuromodulatory effect. This could potentially be compensated for by increasing the ECS current amplitude (see Table 2). Although, as discussed in the next section, work in large animal models is needed to determine the safety of delivering ECS at higher current amplitudes.

### Steps Toward Patient Evaluation

Our results indicate that neuromodulation with concentric-ring ECS may have a number of potentially useful patient applications. However, before these can be fully exploited a number of important steps need to be taken. Our computational model indicated that ECS will generate strong electric fields (>100 V/m) across the skull. Thus, large animal models with skull thicknesses similar to humans should be used to investigate the safety of chronic ECS. Additionally, the redox reactions that take place at an electrode-neuron interface have been reasonably well studied (Yuen et al., 1981; Agnew et al., 1986; Agnew and McCreery, 1990; McCreery et al., 1990). For an ECS electrode similar studies should be undertake for the electrode-bone interface. As we have done here, computational models can be used to study and optimize ECS electrode design. Prototypes of these electrodes must then be manufactured and evaluated in the same large animal models. Finally, ECS needs to be evaluated in patients. Initial evaluations could be done in a noninvasive way using an approach we have recently developed (Khatoun et al., 2018): first a local anesthetic cream is used to numb the scalp; high amplitude stimulation can then be delivered through scalp electrodes to achieve a cortical electric field strength similar to ECS.

## Conclusion

By achieving relatively strong and focused cortical stimulation, in a minimally invasive way, concentric-ring ECS has the potential to offer an alternative neuromodulation therapy for a number of severe neurological and psychiatric disorders.

## Ethics Statement

All procedures were approved by the KU Leuven ethics committee for laboratory experimentation (Project P096/2015).

## Author Contributions

AK: computational modeling, manuscript writing, rats experiments and data analysis. BA: rats experiments, manuscript writing. MM: analysis, manuscript writing, concept. All authors reviewed the manuscript.

## Conflict of Interest Statement

The authors declare that the research was conducted in the absence of any commercial or financial relationships that could be construed as a potential conflict of interest.
