# Supplementary material for: Investigating the Feasibility of Epicranial Cortical Stimulation Using Concentric-Ring Electrodes: A Novel Minimally Invasive Neuromodulation Method
Source: Front Neurosci. 2019 Jul 24;13:773. doi: 10.3389/fnins.2019.00773 (PMC6667561; doi:10.3389/fnins.2019.00773)
Supplement: Supplementary file 1 [file Data_Sheet_1.docx]

Supplementary tables

Table 1: Summary of the recorded data for each rat

| **Rat number** | **Stimulated cortical area** | **Recorded limbs** | **Electrode design** |
| --- | --- | --- | --- |
| Rat 1 | Right hind limb | Right hind limb  Left hind limb | Concentric ring |
| Rat 2 | Left hind limb | Right hind limb  Left hind limb | Concentric ring |
| Rat 3 | Left hind limb | Right hind limb  Left hind limb | Concentric ring |
|  | Right hind limb | Right hind limb  Left hind limb | Concentric ring |
|  | Left fore limb | Left fore limb  Left hind limb | Concentric ring |
|  | Left fore limb | Left fore limb  Left hind limb | Unfocused |
| Rat 4 | Right fore limb | Right hind limb  Right fore limb | Concentric ring |
|  | Right fore limb | Left fore limb  Right fore limb | Concentric ring |
|  | Right hind limb | Right hind limb  Left hind limb | Concentric ring |
|  | Right hind limb | Right hind limb  Right fore limb | Concentric ring |
|  | Right hind limb | Right hind limb  Right fore limb | Unfocused |
| Rat 5 | Right hind limb | Right hind limb  Right fore limb | Concentric ring |
|  | Right hind limb | Right hind limb  Right fore limb | Unfocused |
|  | Left hind limb | Right hind limb  Right fore limb | Concentric ring |
|  | Left hind limb | Right hind limb  Right fore limb | Unfocused |
| Rat 6 | Right hind limb | Right hind limb | Concentric ring |

Supplementary Figures


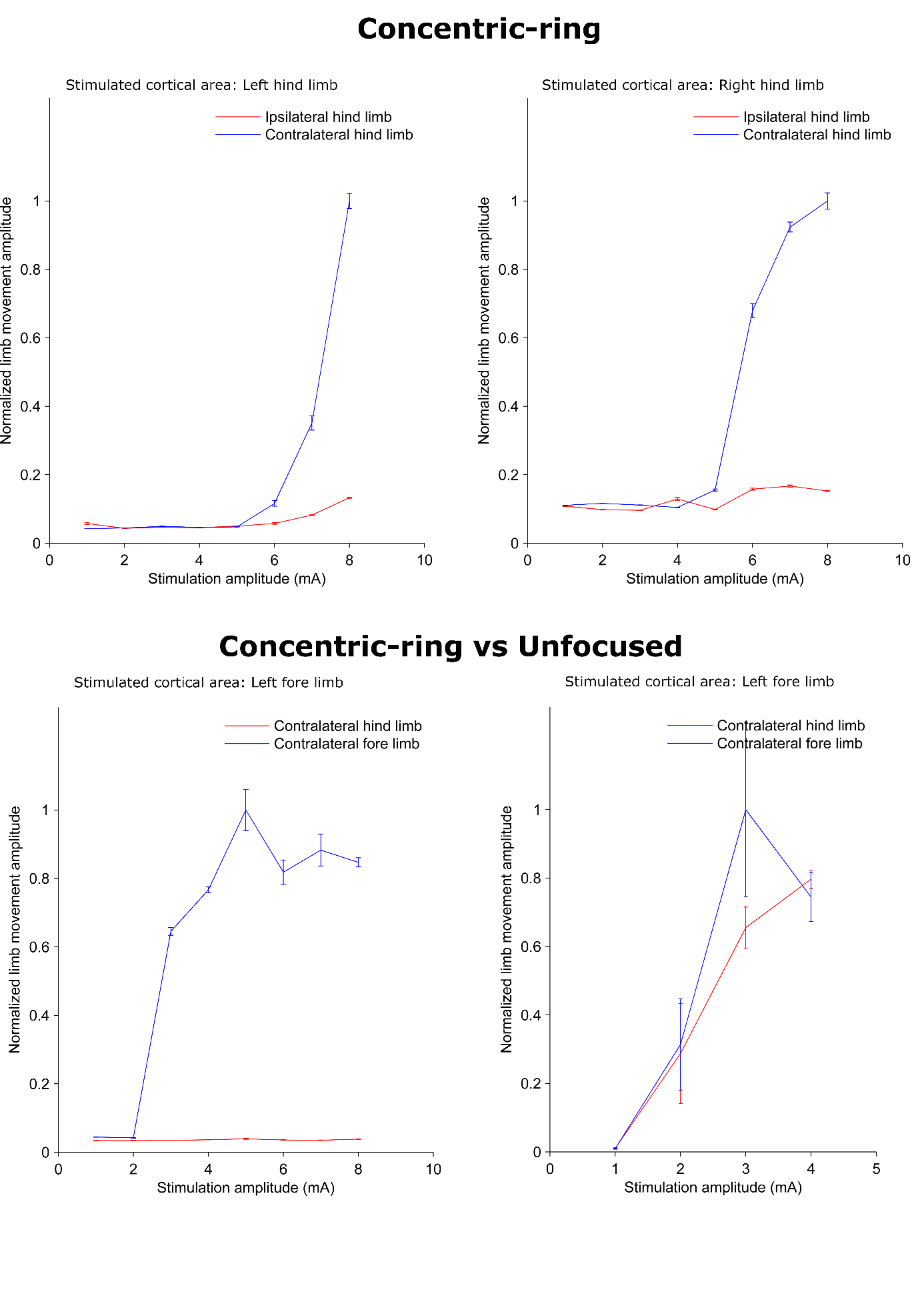


Figure 1: Results obtained from rat number three. The upper row shows the amplitudes of both hind limb movements as a function of stimulation amplitude when the stimulation was targeting the left (upper left panel) and the right hind limbs (upper right panel). The results show a selective movement of the limb contralateral to the side of stimulation. The lower row compares the movement of both fore limbs when concentric-ring (lower left panel) and unfocused (lower right) electrode were used to target the left fore limb cortical region. The results shows a more selective and focused stimulation using concentric-ring electrode. These results indicate that concentric-ring electrode can provide a focused stimulation.


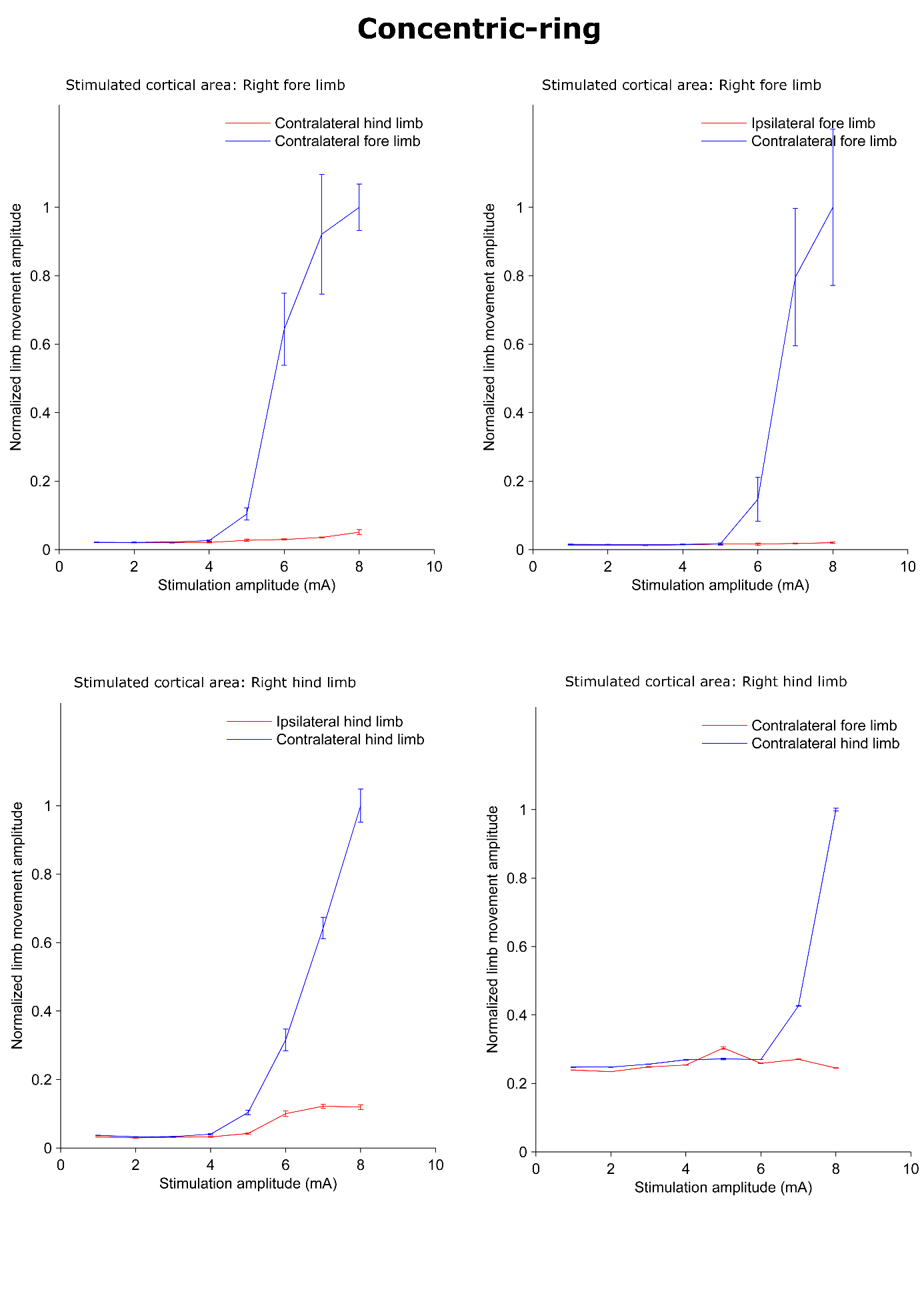


Figure 2: Results obtained from rat number four. The upper row compares the movement of right fore limb to that of the right hind limb (upper left panel) and the right fore limb to that of the left fore limbs (upper right panel) when the stimulation was targeting the right fore limb. The results show a selective movement to the right fore limb. The lower row shows similar results but for the right hind limb, by comparing the movement to that of the left hind limb (lower left panel) and the right fore limb (lower right panel). These results indicate an interhemispheric and intrahemispheric selective stimulation when using a concentric ring electrode. Data comparing concentric-ring to unfocused stimulation in this rat can be found in Fig.2 of the main manuscript.


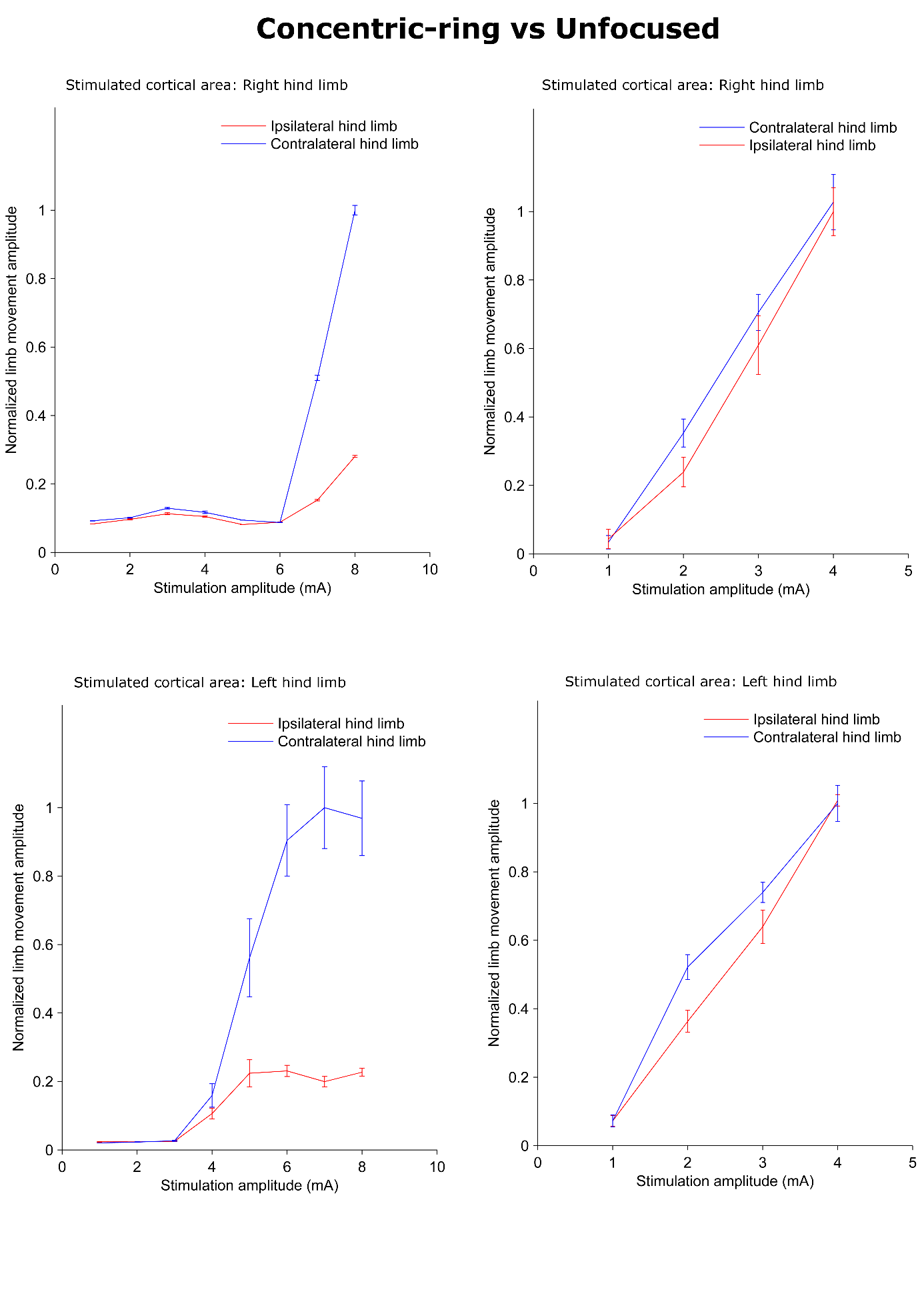


Figure 3: Results obtained from rat number five. The upper row compares the movement of both hind limbs when concentric-ring (upper left panel) and unfocused (upper right panel) electrode were used to target the right hind limb cortical region. The results shows a more selective and focused stimulation using concentric-ring electrode. The lower row shows similar results but this time when the left hind limb was targeted. These results indicate that concentric-ring electrode can provide focused stimulation to the targeted region.


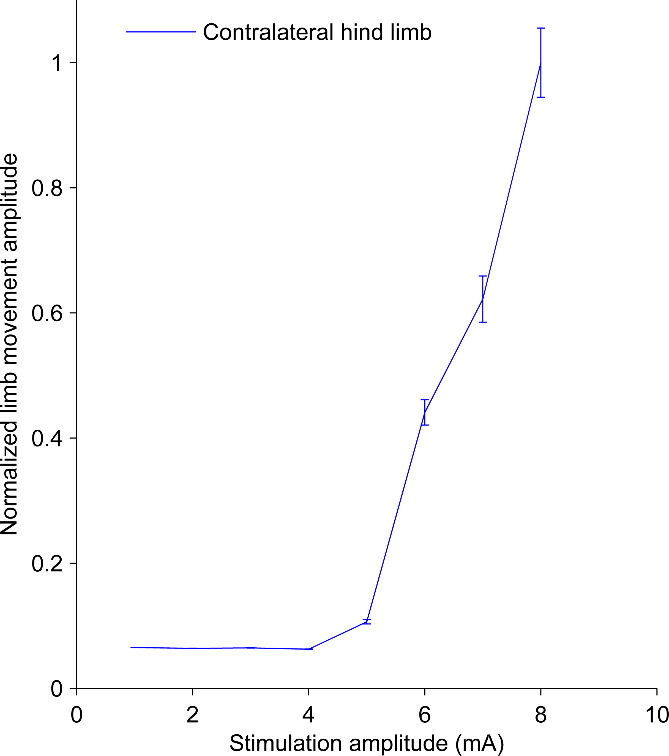


Figure 4: Results obtained from rat number six. The plot shows an increase in the right hind limb when the stimulation amplitude was increased. Movement in the ipsilateral hind limb was not recorded in this rat.
